# The Impact of Pet Health Insurance on Dog Owners’ Spending for Veterinary Services

**DOI:** 10.3390/ani10071162

**Published:** 2020-07-09

**Authors:** Angelica Williams, Brian Williams, Charlotte R. Hansen, Keith H. Coble

**Affiliations:** 1 Genetic Program, DNA Genetics, Columbus, NE 68601, USA; angelicasw.phd@gmail.com; 2 Economic Development Department, Nebraska Public Power District, Columbus, NE 68602, USA; b.r.williamsphd@gmail.com; 3 Veterinary Economics Division, American Veterinary Medical Association, Schaumburg, IL 60173, USA; 4 Department of Agricultural Economics, Mississippi State University, Mississippi State, MS 39762, USA; coble@agecon.msstate.edu

**Keywords:** pet health insurance, pet ownership, access to care, OLS, negative binomial regression, multinomial logistic regression

## Abstract

**Simple Summary:**

Affordability to pay for veterinary services is a challenge for some pet owners. The presence of pet health insurance is one tool that can help alleviate this burden. This study surveyed dog owners, seeking out whether the presence of pet health insurance was a factor in dog owners spending more at the veterinarian, as well as having increased visits to the veterinarian. Results show the presence of pet health insurance had a positive association with spending at the veterinarian, but not with veterinarian visits.

**Abstract:**

The U.S. pet population is increasing, but access to veterinary care continues to be a concern. One method of alleviating barriers that prevent access to care is the presence of pet health insurance for a pet. Dog owners were surveyed to see the impact of pet health insurance on dog owners’ visits and expenditures at the veterinarian. Using several models, it was found that pet health insurance had a significant and positive impact on the amount spent at the veterinarian. Other dog and dog owner characteristics were found significant in impacting expenditures and visits at the veterinarian. Findings from this study can help address the accessibility issue facing Americans across the country in obtaining affordable pet care. This research is the first which seeks to identify the driving factors behind dog owners’ choices regarding health care for their dogs.

## 1. Introduction

Pet owners have elevated their pets to an unprecedented level of companionship, treating the pet as a family member, instead of being viewed as property [1,2,3,4,5,6,7]. In the United States there are an estimated 63.4 million households that own at least one dog, and 42.7 million households that own at least one cat [8]. In a pet owner survey by Packaged Facts, 95% of dog owners and 94% of cat owners agreed (strongly or somewhat) that they considered their dogs or cats to be a part of the family [9]. This is even more so among millennials. In a survey by Weave of 532 millennials, almost 90% of this cohort would put their animal’s life before theirs, and over 90% of millennials care about their pets’ health equally to their own health [10]. With this high reverence for pets, and the willingness to care for a pet’s wellbeing, the need for pet healthcare is evident. For humans, the U.S. health-care industry has medical insurance to help cover visits and treatments for human healthcare. It is also the same for the pet care industry, but currently a lot less regulated [11]. Pet health insurance, like human health insurance, helps pay for the costs of treatments that are difficult to foresee in the future [11]. Policy holders pay a small, known monthly premium to avoid significant financial burdens in the future. However, the presence of an insurance product can sometimes change a consumer’s behavior, such as increasing the number of times one visits the doctor or admitting themselves into a hospital [12]. It is also seen that pet owners with pet health insurance have a higher tendency to spend more towards the end of a pet’s life (similar to humans with health insurance) [11]. Veterinarians and the veterinary care team can take on an important role in showing the benefits of pet health insurance for the owner as an option for payment, but also benefiting the veterinarian and practice as well.

In this article we employ several models looking at different characteristics of dog and dog owners to see what impact the characteristics have on a dog owner’s decision to visit and spend more at the veterinarian, as well as decide between euthanasia or choosing for more expensive treatment options. In particular, the presence of pet health insurance is of interest because it can help pet owners afford veterinary expenses that they may not otherwise be able to afford at the onset of a pet crisis, and the veterinarian is able to give the best care the pet needs without economic limitations from the owner. This research is the first which seeks to identify the driving factors behind dog owners’ choices regarding health care for their dogs. 

### 1.1. Pet Health Insurance Awareness

Pet health insurance has been around in the United States since 1980, but the adoption of pet health insurance as a method to pay for veterinary services has been slow to become accepted, unlike other countries such as Canada, the United Kingdom, and Sweden [13]. In fact, the United Kingdom has a robust pet health insurance industry, and even China has seen growth in pet health insurance [14,15]. A key reason for the low adoption rate in the United States is the lack of awareness and understanding of pet health insurance among pet owners; however, as more people become aware, the adoption rate of pet health insurance will continue to grow. The adoption rates of pet health insurance in the United States is at 2.3% of dogs (1.7% in 2017), and 0.4% of cats (0.3% in 2017), according to the North American Pet Health Insurance Association (NAPHIA) [16]. That equates to about 2.15 million pets that were insured in the U.S. at the end of 2018, an 18% increase from the prior year [16]. Since 2014, the compound annual growth rate in the U.S. is 14.7% and this number continues to grow [16].

One way to raise awareness of pet health insurance in the United States as a payment method is through the veterinarian. Past research has shown when pet owners were asked what sources of information for pet care were most important to them, 70% of pet owners said the veterinarian was the most important [17]. This is a common theme, as a 2016 nationwide pet health insurance study by NAPHIA found that over half of pet owners would purchase pet health insurance if their regular veterinarian recommended it [16], and in a consumer preferences survey conducted by the American Veterinary Medical Association in 2015 [18], 65% of respondents purchased pet health insurance because a veterinarian recommend it. In addition, 56% of dog owners and 42% of cat owners reported likely, or extremely likely, to purchase pet health insurance in the future if it were recommended by their veterinarians, and 40% of dog owners and 41% of cat owners prefer to buy pet health insurance from a veterinary provider. In Coe, Adams, and Bonnett [19], pet owners were interested in learning about pet health insurance from their veterinarian but received little information about pet health insurance as an option to help defray the cost of veterinary care.

### 1.2. Veterinary Expenditures and Visits

While dog ownership has gained popularity, with over 38% of households owning dogs, the number of visits to the veterinarian, for both dogs and cats, have seen a decline over the years. For example, in 2011, dog-owning households visited the veterinary clinic or hospital about 130.4 million times, an increase of 9% from 2006; by 2016, total visits had fallen to 123.3 million [3]. While there has been a decline in the number of visits, the average veterinary expenditure per visit has risen from $138 to $161 and has outpaced U.S. inflation; this is due to increased labor costs, rising costs of medical equipment and supplies, and more, all contributing to more expensive veterinary care [3]. The 2016 NAPHIA study revealed that pet health insurance increased pet expenditures at the veterinarian, as well as increased the number of visits. Specifically, 29% of dog owners and 81% of cat owners spent more per year on veterinary care [16]. 

### 1.3. Ability to Afford Veterinary Care

While research has looked at factors associated with increased expenditures and visits at a veterinarian, top reasons for a pet owner not bringing their pet to the veterinarian is because the animal was not sick or injured, and because the owner did not have money to pay for it. Interestingly, 5% of pet owners also stated that cost of care was more than they thought it was worth, versus 23% saying they lacked the money to pay for care [3]. In other words, the issue was less about a lack of perceived value and more about inability to afford veterinary care. A 2019 study by the United Veterinary Services Association revealed that 48% of pet owners are not fully satisfied with the veterinary clinic they go to because of cost/price issues; however, this study also showed that over 95% of cat and dog owners were satisfied with the quality of medical care (excluding cost factors) they received for their pets [17]. Owners not having money to pay for a veterinary service is an issue. Based on the roughly 30% of pet owners that do not see a veterinarian at least once a year, and the average expenditure per pet, this translates into $7 billion of veterinary care not being delivered to animals. This is a miss for the economic sustainability of the veterinary practice but also for the livelihood of pets [20]. In these studies, seeking out veterinary services was not a matter of perceived value, but the lack of affordability to pay for those services. One way to alleviate the access to care issue is the presence of pet health insurance. 

Affordability of veterinary care was a challenge for both owners and veterinarians in a focus group study by Coe, Adams, and Bonnett [19]. There was an expectation by pet owners who could and who could not afford expensive veterinary services that veterinarians should offer some type of payment plan to alleviate the upfront cost. From the veterinarian’s point-of-view, working around the client’s budget restrictions is tricky, as they may not be able to offer all the care the pet needs [19]. In another study by Kipperman, Kass, and Rishniw, economic limitations from clients prevented veterinarians from providing the best care for their client’s pets, resulting in euthanizing pets that could otherwise be saved [21]. Not only did economic limitations prevent veterinarians from providing the best care for pets, but veterinarians cited euthanasia as a reason for a large level of burnout within themselves. In addition, only about a quarter of veterinarians surveyed discussed pet health insurance as an available option for cost of care before the pets became sick, noting that not having enough time to talk to their clients about pet health insurance was a reason clients were unaware. In addition, in this study, most respondents agreed that clients knowing about pet health insurance would improve patient care, and in addition, reduce burnout (by improving the satisfaction level of veterinarians) [21].

## 2. Materials and Methods

A quantitative research method based on an online survey of pet owners with and without pet health insurance across the U.S. was used to collect dog and dog owner characteristics to measure the impact on the frequency of veterinary visits and expenditures on veterinary pet healthcare. Prior to implementing the survey, the survey instrument was reviewed and approved by the Institutional Review Board for the Protection of Human Subjects in Research (IRB) at Mississippi State University. A nationwide survey of dog owners with and without pet health insurance was launched in January 2017 via email using Qualtrics survey software (Qualtrics Labs, Inc., Provo, UT, USA). Respondents were selected randomly by Qualtrics’s panels and are representative of the general U.S. population. Responses to the questions were screened for inconsistent, illogical, or straight-lined responses, or in instances where the respondent completed the survey too quickly. Information collected included information on the dog owners’ relationships with their pets (whether there is a strong human–animal bond based on where the pet sleeps at night), the available alternatives—pet health insurance (covers unexpected costs, such as bloodwork, surgeries, or cancer treatments) and/or wellness plans (covers routine veterinary care, such as wellness checkups, vaccines, and spaying and neutering; wellness plans can be a part of pet insurance plans or stand-alone) the dog owner has purchased, owners’ attitudes toward risk (major dog illness in the past, perceived future risk of dog illness, and whether a $1000 veterinary bill would cause financial stress), and factors that influence their decisions like sociodemographic characteristics of the dog owner (education, income, age, gender, employment, pet expenditures) and dog (purebred, age, size of dog). With regards to dog owner income, income greater than $55,000 was used as the cut-off point (i.e., dummy variable) because when the survey was administered the median U.S. household income was approximately $55,000. In theory, individuals who make more than $55,000 would be more willing/able to spend a portion of that income on pets and pet-related expenses as their income allows them to spend beyond essential living expenses (rent, food, gas, etc.). Definitions of variables can be found in Table 1. Table 2 reports the summary statistics for these variables.

**Figure 1:**
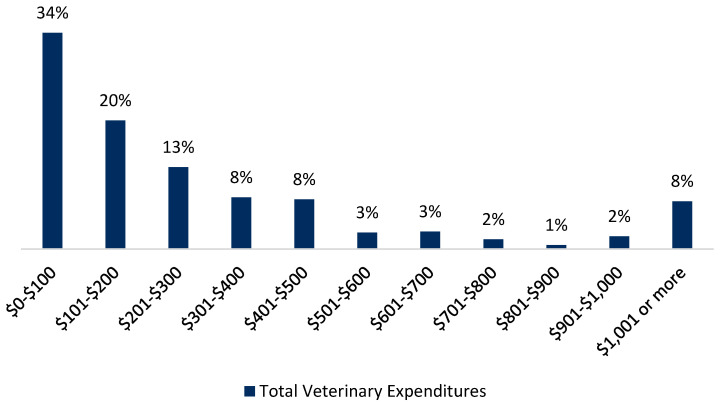
Total veterinary expenditures per pet owner per year.

**Figure 2:**
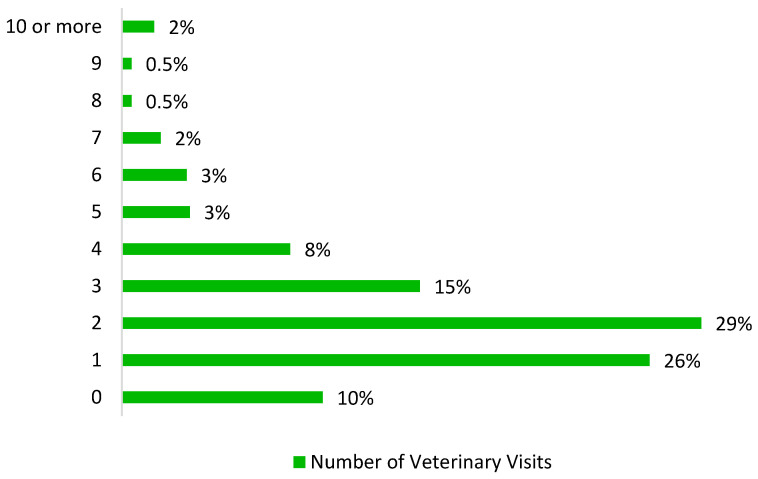
Total veterinary visits per pet owner per year.

Data were analyzed using STATA 16. A least squares regression was utilized to estimate the impact of insurance on pet healthcare expenditures (Figure 1) and a negative binomial regression to estimate the impact of pet health insurance on the number of veterinary visits (Figure 2). Lastly, a multinomial logistic regression was utilized to estimate the impact that pet health insurance and other variables have on a dog owner’s treatment choice when posed with a hypothetical scenario in which their dog faces a major health diagnosis that requires immediate treatment.

The ordinary least squares (OLS) estimation determines the impact of dog and dog owner characteristics on expenditures at the veterinarian office each year. A likelihood ratio test shows that the OLS model is the best fit for the given data. Dog and dog owner characteristics included in the model are whether or not the dog is insured, the presence of a wellness plan, whether the dog is a purebred or a mixed breed, the age of the dog, whether the dog has been spayed or neutered, the size of the dog, the gender and employment status of the dog owner, expenditures on pet food, toys, and other non-healthcare related items, the dog owner’s age, the education level of the dog owner, the income of the dog owner, where the dog sleeps at night, whether an unexpected $1000 vet bill would cause financial stress, whether the dog has had a major illness in the past, and the dog owner’s risk perceptions regarding future illness. The model is expressed as:

*E[Expenditures!] = f (insurance, wellness plan, purebred, dog’s age, spayed/neutered, large breed, owner gender, owner employment status, expenditures on other items, owner’s age, owner’s education, owner’s income, where the dog sleeps, would $1000 bill cause financial stress, major illness in the past, perceived likelihood of major illness in the next year)* (1)

Similar to the ordinary least squares model mentioned above, the negative binomial regression model examines the impact of dog and dog owner characteristics on the number of visits that a dog makes to a veterinarian’s office each year. The negative binomial regression model is ideal for situations in which the dependent variable is a count variable and is commonly used to compute the probability of observing a number of “successes” if the process is repeated. Our model can be expressed as:

*E[Visits!]= f(insurance, wellness plan, purebred, dog’s age, spayed/neutered, large breed, owner gender, owner employment status, expenditures on other items, owner’s age, owner’s education, owner’s income, where the dog sleeps, would $1000 bill cause financial stress, major illness in the past, perceived likelihood of major illness in the next year)* (2)

where *Total*! is the total number of recommended practices adopted by producer i and the independent variables are categorical measures of producer characteristics. The “base producer” is represented at the means of the demographic variables.

Finally, a multinomial logistic regression was utilized to estimate the impact that pet health insurance and other variables have on a pet owner’s treatment choice when posed with a hypothetical scenario in which their dog faces a major health diagnosis that requires immediate treatment. Multinomial logistic models are ideal for modeling a consumer’s choice when there are more than two options. The respondents were given four treatment options: Euthanasia, spend $1000 for survival but moderate health problems for the remainder of your pet’s life, spend $3000 for survival but minor health problems for the remainder of your pet’s life, or spend $10,000 for a full recovery and no further health problems. The base response in this model is euthanasia and the coefficients can be interpreted as the marginal impact that each variable has on the likelihood that a pet owner chooses each respective treatment over euthanasia. As with the above models, the explanatory variables include whether or not the dog is insured, the presence of a wellness plan, whether the dog is a purebred or a mixed breed, the age of the dog, whether the dog has been spayed or neutered, the size of the dog, the gender and employment status of the dog owner, expenditures on pet food, toys, and other non-healthcare related items, the dog owner’s age, the education level of the dog owner, the income of the dog owner, where the dog sleeps at night, whether an unexpected $1000 vet bill would cause financial stress, whether the dog has had a major illness in the past, and the dog owner’s risk perceptions regarding future illness. The model is defined as:

*Pr[Yi = K]= f(insurance, wellness plan, purebred, dog’s age, spayed/neutered, large breed, owner gender, owner employment status, expenditures on other items, owner’s age, owner’s education, owner’s income, where the dog sleeps, would $1000 bill cause financial stress, major illness in the past, perceived likelihood of major illness in the next year)* (3)

where Pr[Yi = K] is the probability that pet owner i chooses K as a treatment option.

## 3. Results and Discussion 

**Figure 3:**
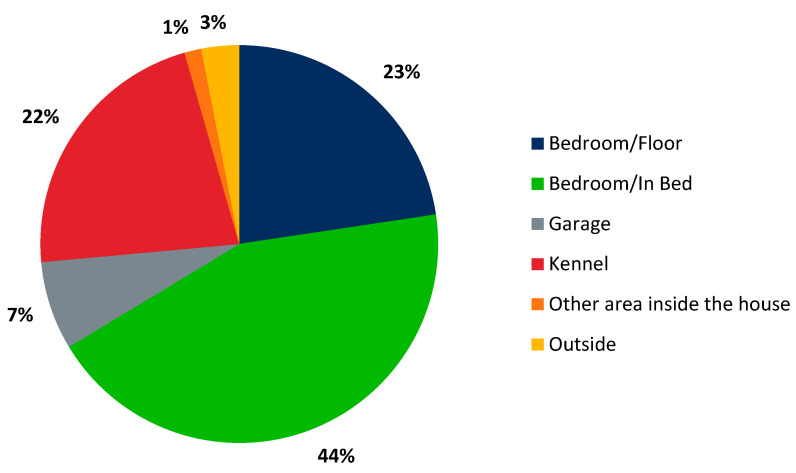
Where the dog sleeps at night: The majority of pet owners allow their pet to sleep in the bedroom.

In total, there were 654 total observations, 442 dog owners with pet health insurance (67.6%) and 212 without pet health insurance (32.4%). Almost half of dog owners said they participated in a preventative care plan that helps pay for wellness visits. Of those insured, three-quarters of pet owners had a preventative care plan for their dog. There were 36.5% male dog owners and 63.5% female dog owners. Most of the dog owners were employed, at least high school educated, and 2/3 of respondents had an income of $55,000 or greater. The average age of the dog was around 5 years old. Almost half were purebred and less than half were spayed or neutered. Most dog owners had small breed dogs. Two-thirds of pet owners stated that their dog slept in the bedroom (Table 2). More specifically, 44% of dogs slept in the owner’s bed, 23% on the bedroom floor, 22% of dogs were kenneled at night, 7% stayed in the garage, 3% of dogs stayed outside, and 1% stayed in some other area of the house (Figure 3). When it came to illness history of the dog, 22.8% of dog owners stated their dog had a major illness in the past, and 20.1% of dog owners stated they perceived their dog to come down with a major illness in the next year. A little over half of respondents stated that a $1000 veterinary bill would cause financial stress (Table 2).

As reported in Table 3, results from our OLS model indicate that our main variable of interest, insurance, has a statistically significant and positive impact on pet healthcare expenditures. Individuals who have pet health insurance spent an average of $211 more than those without pet health insurance. However, it should be noted that there are several other drivers of pet owners’ expenditures in addition to pet health insurance. For example, owners that had an income greater than $55,000 spent an average of $164 more than owners with less than $55,000. Pet owners with more than $55,000 could be more willing/able to increase expenditures on pets and pet-related expenses as their disposable income allows them to spend beyond essential living expenses such as rent, food, and gas. A pet owner’s expenditures on other areas related to their pets (i.e., food, toys, grooming) also has a strong influence on healthcare expenditures—if an owner spends more on other expenditures with their pet they could be likely to have disposable income to afford more veterinary care when needed. Where the pet sleeps at night is also statistically significant, with pet owners who allow their pet to sleep in the bedroom spending an average of $105 more compared to owners who do not allow their pet to sleep in the bedroom. This is confirmed in the literature where pet owners with a stronger human–animal bond spend more on their pet [1,2,3,4,5,6,7]. Owning a large breed of dog, past experiences regarding a pet’s health circumstances, and the pet owner’s perceived risk of future health problems also drive expenditures up. Large breed dogs tend to cost more to upkeep, and if an owner has had pet health problems in the past and think there will be an issue in the future, on average they will spend more than owners that have pets that are generally healthy. There are also several other factors that need to be examined. For example, while our findings show that on average pet health insurance does impact expenditures, we did not control for any of the attributes within an insurance plan. It is possible that attributes such as deductible level or reimbursement rates of the insurance plan may have an impact on overall expenditures. With that being said, there may be pet health insurance plans with certain combinations of attributes that do not impact expenditures. We also checked for interactions on the insurance plan attributes, and found no statistical significance for deductible, payout limits, etc.

Although our research indicates that our variable of interest, insurance, has a positive impact on expenditures, as shown in Table 3, we did not find a statistically significant impact from pet health insurance on the number of visits to a veterinary office once all other factors had been taken into account (Table 4). While owners with pet insurance spend more at the veterinarian, they may be likely to utilize services in one visit, as opposed to spreading it throughout the year on multiple visits. Among the factors that do impact the number of visits, we found that a wellness plan, expenditures on non-health related areas, where the pet sleeps at night, past illnesses, and perceived risk of future illness had a positive impact. Factors such as a dog’s age and lower education levels had a negative impact on the number of visits. It should be noted, however, that the coefficients from the negative binomial count model are not directly interpretable. 

As a result, we also calculated the incidence rate ratio (IRR) for each variable, which can be more directly interpreted. Using the IRR, we found that those with a wellness plan visited the veterinarian’s office 1.2 times, or 20% more, than those who did not have a wellness plan. A wellness plan can encourage a pet owner to obtain preventive care such as multiple wellness checkups, vaccines, nail trims, flea and heartworm prevention, etc. throughout the year. In addition, veterinary visits increased 1.5% more for every $1000 a pet owner spent on food, grooming, toys, or other “treats” for their pet. A pet who sleeps in the owner’s bedroom also had about 20% more visits than a pet who sleeps outside the owner’s bedroom. When it comes to risk, owners that had their dog ill in the past visited the veterinarian 18% more compared to pets that did not have a major illness in the past. There was little difference in the IRR for pet owners that perceived their dog would have a major illness in the next year (Table 5).

We also posed dog owners with a hypothetical situation in which their dog faces a major health diagnosis that requires immediate treatment. The respondents were given four treatment options: 1 = euthanasia, 2 = spend $1000 for survival but moderate health problems for the remainder of your dog’s life, 3 = spend $3000 for survival but minor health problems for the remainder of your dog’s life, or 4 = spend $10,000 for a full recovery and no further health problems. The base choice is 1, euthanasia. We found that those who had pet health insurance were more likely to choose the $1000 treatment over euthanasia than those without insurance, but insurance had no impact on the decision to choose the two more expensive treatment options (Table 6). This seems to suggest that while pet health insurance may drive dog owners to seek necessary treatment to save their dog’s life, it does have its limitations when it comes to more expensive treatment options. The cost of more expensive treatment options could reflect the severity of an illness or injury, which would have associated perceptions on suffering and welfare requirements in which owners will choose to euthanize rather than spend more at the veterinarian and see their pet suffer. Not surprisingly, as a dog gets older pet owners are less likely to choose any of the treatments over euthanasia. We suspect that after a dog reaches a certain age, the tradeoff between an expensive treatment and the additional amount of time that the pet owner has to spend with their dog becomes much less favorable.

Income also had a significant impact on the dog owner’s treatment choice. Individuals with a higher income were much more likely to choose the $3000 and $10,000 treatments than those with an income of less than $55,000, suggesting that those with more expendable income were willing to invest more into their dog’s health and well-being. An owner’s relationship with their dog also appears to have an impact on their willingness to select higher-cost treatments. Those who allow their dogs to sleep in their bedroom have a statistically significant and positive correlation with more expensive treatment options and were more likely to select the $3000 and $10,000 treatments than euthanasia, and just barely missed having a statistically significant result for the $1000 treatment. A dog that sleeps in the owner’s bedroom (whether on the floor or in the bed) is assumed to be correlated with how close an owner is with their dog (as member of the family rather than a pet), as the human–animal bond increased, pet expenditures also increased [1,3,6]. We have also seen in prior results that the more disposable income an owner has the more they spend at the veterinarian’s office. 

There was also a correlation with whether a $1000 veterinary bill would cause financial stress. If respondents said yes, a $1000 veterinary bill would cause financial stress, they were less likely to select the $10,000 treatment and choose euthanasia (the $1000 and $3000 treatments were not significant). Again, income was a driving factor among pet owners, and income can prevent accessibility to veterinary care (Table 6).

## 4. Conclusions

From this research we saw that pet health insurance increased the amount spent at the visit, although frequency of visits was not significantly impacted by having pet health insurance. Pet owners may be spending more at the veterinarian and getting more of the services they need in one visit, because pet health insurance has allowed them to purchase with ease of mind. The challenge of affordability is not going away. Having conversations with the pet owner on the many tools (insurance, wellness plans, etc.) to support veterinary care is key. Veterinarians have a substantial impact on an owner’s decisions with regard to their pet’s care. Forty-nine percent of dog or cat owners would be likely or extremely likely to purchase insurance if veterinarians recommended pet health insurance as a method of payment, and owners would be most willing to purchase a pet health insurance policy directly from their veterinarian, with 40% of respondents indicating that this would be their preferred provider [22]. Communicating the importance of preventive and wellness care to pet owners is critical to closing the care gap. Educating clients on alternative methods of payment can increase the level of care pet patients receive, improving their overall health, quality of life, and life expectancy. 

This research is the steppingstone for future work on pet health insurance. Future work should include analyzing to what extent pet health insurance impacts consumer behavior, the importance of plan attributes in a pet health insurance plan (the positive or negative impact that different deductibles, reimbursement rates, and conditions have within a plan), and what role a wellness plan has within a pet health insurance policy and its impact on visits, expenditures, and overall pet health. Additional work could include focusing on a veterinarians’ willingness to provide or promote a bundled wellness/insurance product, and their concerns surrounding the products in terms of issues that may prevent them from offering it, and/or costs involved for them. 

## Figures and Tables

**Table 1:** Variables and variable definitions.

Variables	Definitions
Insured	Insured = 1 if the dog is insured and 0 otherwise.
Wellness plan	Wellness plan = 1 if there is a presence of a wellness plan and 0 otherwise.
Purebred	Purebred = 1 if the dog is a purebred and 0 if a mixed breed.
Dog’s age	Age of dog.
Fixed	Fixed = 1 if the dog has been spayed or neutered and 0 otherwise.
Large breed	Large breed = 1 if the size of the dog is a large breed and 0 otherwise.
Male owner	Male owner = 1 if the dog owner is male and 0 if the owner if a female.
Owner unemployed	Owner unemployed = 1 if the dog owner is unemployed and 0 otherwise.
Expenditures on other	Dollars spent on pet food, toys, and other non-healthcare related items.
Owner age	The age of the dog owner.
High school education	High school education = 1 if the dog owner has at least a high school diploma and 0 otherwise.
Income > $55,000 *	Income >$55,000 = 1 if the dog owner has a household income greater than $55,000 and 0 otherwise.
Sleep in bedroom	Sleep in bedroom = 1 if the dog sleeps in the bedroom (on the floor or in the bed) and 0 otherwise.
$1000 bill causes financial stress	Stress1000 = 1 if an unexpected $1000 vet bill would cause financial stress and 0 otherwise.
Major illness in the past	Pastmajor = 1 if the dog has had a major illness in the past.
Perceived likelihood of major illness in the next year	Majorillnessprob1000 = 1 if the dog owner perceives their dog to get ill in the future and 0 otherwise.

* Income greater than $55,000 was used because when the survey was administered the median U.S. household income was approximately $55,000. In theory, individuals who make more than $55,000 would be more willing/able to spend a portion of that income on pets and pet-related expenses as their income allows them to spend beyond essential living expenses (rent, food, gas, etc.).

**Table 2:** Summary statistics of variables used in the econometric analysis. Frequency and percentages are provided for categorical variables, and median, standard deviation, minimum, and maximum are provided for continuous variables.

	Frequency	Percentage	Median	Std. Dev.	Min.	Max.
**Insured**						
*Yes*	442	67.6%				
*No*	212	32.4%				
**Wellness plan**						
*Yes*	322	49.2%				
*No*	332	50.8%				
**Purebred**						
*Yes*	316	48.3%				
*No*	338	51.7%				
**Dog’s age**	654		4.7	3	0	10
**Fixed**						
*Yes*	275	42.1%				
*No*	379	57.9%				
**Large breed**						
*Yes*	117	17.9%				
*No*	537	82.1%				
**Male owner**						
*Yes*	239	36.5%				
*No*	415	63.5%				
**Owner unemployed**						
*Yes*	98	15.0%				
*No*	556	85.0%				
**Expenditures on other**	654		$45.80	$211.00	$0.00	$3200.00
**Owner age**	654		40.4	14.3	18	79
**High school education**						
*Yes*	526	80.4%				
*No*	128	19.6%				
**Income > $55,000**						
*Yes*	430	65.8%				
*No*	224	34.2%				
**Sleep in bedroom**						
*Yes*	434	66.4%				
*No*	220	33.6%				
**$1000 bill causes financial stress**						
*Yes*	337	51.5%				
*No*	317	48.5%				
**Major illness in the past**						
*Yes*	149	22.8%				
*No*	505	77.2%				
**Perceived likelihood of major illness in the next year**			20.1%	23.1%	0%	100%

* This includes pet food, toys, and all other non-health care related expenditures.

**Table 3:** Ordinary least squares (OLS) regression results with total expenditures as the dependent variable.

Variable	Parameter Estimate	Std. Error	*p*-Value
Intercept	−230.13	160.94	0.15
Insured	211.16	103.90	0.04 **
Wellness	−162.55	108.79	0.13
Purebred	−4.80	61.54	0.93
Dog’s age	4.56	8.02	0.57
Fixed	78.32	56.98	0.17
Large breed	232.26	82.22	0.005 ***
Male owner	5.63	68.58	0.93
Owner unemployed	3.96	67.22	0.95
Expenditures on other *	0.16	0.04	0.0001 ***
Owner age	1.33	1.69	0.43
High school education	−24.04	50.11	0.63
Income > $55,000	164.19	52.10	0.002 ***
Sleep in bedroom	105.26	48.60	0.03 **
$1000 bill causes financial stress	59.39	63.99	0.35
Major illness in the past	161.95	76.12	0.03 **
Perceived likelihood of major illness in the next year	4.23	1.45	0.004 ***

* This includes pet food, toys, and all other non-health care related expenditures. *** and ** indicate significance at the 1% and 5% levels, respectively.

**Table 4:** Negative binomial count model with number of visits as the dependent variable.

Variable	Parameter Estimate	Std. Error	*p*-Value
Intercept	0.5984	0.1503	<0.0001 ***
Insured	−0.1055	0.0904	0.2434
Wellness	0.1790	0.0853	0.0359 **
Purebred	0.0700	0.0636	0.2717
Dog’s age	−0.0371	0.0109	0.0007 ***
Fixed	0.0175	0.0656	0.7895
Large breed	0.0674	0.0775	0.3851
Male owner	−0.0885	0.0686	0.1972
Owner unemployed	0.0185	0.0924	0.8412
Expenditures on other *	0.0002	0.0000	<0.0001 ***
Owner age	−0.0013	0.0023	0.5761
High school education	−0.2379	0.0895	0.0079 ***
Income > $55,000	0.0576	0.0677	0.3947
Sleep in bedroom	0.1869	0.0691	0.0068 ***
$1000 bill causes financial stress	0.1123	0.0643	0.0806
Major illness in the past	0.1653	0.0730	0.0235 **
Perceived likelihood of major illness in the next year	0.0048	0.0013	0.0004 ***
Dispersion	0.1628	0.0304	

* This includes pet food, toys, and all other non-health care related expenditures. *** and ** indicate significance at the 1% and 5% levels, respectively.

**Table 5:** Incidence rate ratios for negative binomial count model with number of visits as the dependent variable.

Variable	Parameter Estimate	Std. Error	*p*-Value
Insured	0.8999	0.0814	0.2434
Wellness	1.1960	0.1020	0.0359 **
Purebred	1.0725	0.0683	0.2717
Dog’s age	0.9636	0.0105	0.0007 ***
Fixed	1.0177	0.0668	0.7895
Large breed	1.0697	0.0830	0.3851
Male owner	0.9153	0.0628	0.1972
Owner unemployed	1.0187	0.0941	0.8412
Expenditures on other *	1.0002	0.0000	<0.0001 ***
Owner age	0.9987	0.0023	0.5761
High school education	0.7883	0.0706	0.0079 ***
Income > $55,000	1.0593	0.0717	0.3947
Sleep in bedroom	1.2055	0.0833	0.0068 ***
$1000 bill causes financial stress	1.1189	0.0719	0.0806
Major illness in the past	1.1797	0.0861	0.0235 **
Perceived likelihood of major illness in the next year	1.0048	0.0013	0.0004 ***

* This includes pet food, toys, and all other non-health care related expenditures. *** and ** indicate significance at the 1% and 5% levels, respectively.

**Table 6:** Multinomial logit results where pet owners were posed with a hypothetical situation in which their pet faces a major health diagnosis and are given four treatment options.

Parameter	Choice *	Estimate	Standard Error	*p*-Value
Intercept	2	−0.8917	0.8374	0.2869
Intercept	3	0.4850	0.6956	0.4857
Intercept	4	0.5342	0.7034	0.4476
Insured	2	2.0186	0.4424	<0.0001 ***
Insured	3	−0.0392	0.3187	0.9022
Insured	4	0.3815	0.3226	0.2370
Purebred	2	0.3762	0.3590	0.2946
Purebred	3	0.0283	0.3228	0.9302
Purebred	4	0.3194	0.3224	0.3218
Dog’s age	2	−0.1390	0.0594	0.0192 **
Dog’s age	3	−0.1022	0.0516	0.0477 **
Dog’s age	4	−0.1466	0.0523	0.0051 ***
Fixed	2	−0.6454	0.3764	0.0864
Fixed	3	0.0214	0.3297	0.9483
Fixed	4	−0.0593	0.3311	0.8578
Large breed	2	−1.0110	0.4515	0.0251 **
Large breed	3	−0.2669	0.3637	0.4631
Large breed	4	−0.3115	0.3645	0.3928
Male owner	2	−0.6345	0.3829	0.0975
Male owner	3	-0.2122	0.3305	0.5207
Male owner	4	−0.1984	0.3314	0.5494
Owner unemployed	2	0.1644	0.5055	0.7451
Owner unemployed	3	0.1107	0.4490	0.8052
Owner unemployed	4	−0.2872	0.4623	0.5345
Expenditures on other ****	2	−0.00028	0.000234	0.2294
Expenditures on other ****	3	−0.00002	0.000191	0.8978
Expenditures on other ****	4	0.00003	0.000188	0.8728
Owner age	2	0.0201	0.0131	0.1243
Owner age	3	0.0193	0.0116	0.0946
Owner age	4	0.0134	0.0117	0.2508
High school education	2	0.1640	0.4915	0.7386
High school education	3	0.3493	0.4355	0.4225
High school education	4	0.6478	0.4356	0.1370
Income > $55,000	2	0.2320	0.3742	0.5353
Income > $55,000	3	0.6495	0.3394	0.0557 **
Income > $55,000	4	0.6654	0.3405	0.0507 **
Sleep in bedroom	2	0.7054	0.3659	0.0539
Sleep in bedroom	3	1.4580	0.3273	<.0001 ***
Sleep in bedroom	4	1.6487	0.3296	<.0001 ***
$1000 bill causes financial stress	2	−0.2457	0.3692	0.5058
$1000 bill causes financial stress	3	−0.5639	0.3282	0.0858
$1000 bill causes financial stress	4	−0.7619	0.3291	0.0206 **
Major illness in the past	2	−0.0846	0.4486	0.8504
Major illness in the past	3	−0.2272	0.4055	0.5753
Major illness in the past	4	0.3739	0.3959	0.3449
Perceived likelihood of major illness in the next year	2	0.00354	0.00699	0.6125
Perceived likelihood of major illness in the next year	3	−0.00976	0.00670	0.1453
Perceived likelihood of major illness in the next year	4	−0.0151	0.00679	0.0258 **

* Base choice is 1. Choices are defined as: 1 = euthanasia, 2 = spend $1000 for survival but moderate health problems for the remainder of your pet’s life, 3 = spend $3000 for survival but minor health problems for the remainder of your pet’s life, and 4 = spend $10,000 for a full recovery and no further health problems. *** and ** indicate significance at the 1% and 5% levels, respectively. **** This includes pet food, toys, and all other non-health care related expenditures.
